# Decreased clinical and functional outcomes following reverse total shoulder arthroplasty for proximal humerus fractures compared to rotator cuff arthropathy: a systematic review and meta-analysis

**DOI:** 10.1016/j.xrrt.2026.100691

**Published:** 2026-02-06

**Authors:** Shahabeddin Yazdanpanah, Bryan T. Soth, Joshua R. Eskew, Malik Dancy, Michael C. Fu, Samuel A. Taylor, Joshua S. Dines, David M. Dines, Lawrence V. Gulotta, Christopher M. Brusalis

**Affiliations:** a College of Medicine, Northeast Ohio Medical University, Rootstown, OH, USA; b Hospital for Special Surgery, New York, NY, USA

**Keywords:** Reverse total shoulder arthroplasty, Proximal humerus fracture, Rotator cuff arthropathy, Revision, PROM, ASES, Range of motion, Dislocation

## Abstract

**Background:**

Comparative studies of reverse total shoulder arthroplasty (rTSA) for proximal humerus fractures (PHFs) versus rotator cuff arthropathy (RCA) have yielded conflicting findings and lack comprehensive synthesis to guide perioperative counseling. This study aimed to systematically compare clinical and functional outcomes between patients undergoing rTSA for PHFs and RCA.

**Methods:**

A PROSPERO-pre-registered systematic review and meta-analysis queried PubMed, CINAHL, MEDLINE, and Web of Science on July 25, 2025, for studies comparing PHF- versus RCA-indicated rTSA. Study quality was assessed using the Methodological Index for Non-Randomized Studies scale. Extracted variables included patient demographics, survey scores, range of motion, and revisions. Patient-reported outcome measures and functional outcomes were compared between patients undergoing rTSA for PHFs and RCA.

**Results:**

Eleven observational studies encompassing 6,698 patients (1,832 PHF; 4,866 RCA) met criteria; overall evidence quality was moderate. Compared with RCA, PHF patients had lower American Shoulder and Elbow Surgeons scores (mean difference −5 points; *P* = .045) and reduced forward flexion (−14°; *P* < .001) and abduction (−17°; *P* < .001). Pain scores were similar (visual analog scale; *P* = .62). Overall revision risk did not differ, but PHF patients had a higher revision risk from dislocation (risk ratio 1.59; *P* = .04). Implant survivorship appeared similar across groups.

**Conclusion:**

RTSA for PHF yields slightly lower shoulder function, range of motion, and a higher dislocation-related revision risk compared with RCA; though, absolute differences were modest. These findings support nuanced preoperative counseling, highlighting opportunities to optimize PHF-specific surgical strategies.

Modern reverse total shoulder arthroplasty (rTSA), first introduced by Paul Grammont in the 1980s, has been transformational for the management of advanced shoulder disease.^11^ Originally indicated for rotator cuff arthropathy (RCA), rTSA reverses the anatomical configuration of the shoulder joint to enhance range of motion (ROM) and compensatory muscle engagement when otherwise unamenable.^8^^,^^11^^,^^28^^,^^30^ These clinical advantages have fueled rapid adoption of rTSA, rising from 32% of primary shoulder arthroplasty procedures to nearly 70% by 2020.^38^^,^^53^ rTSA has expanded beyond RCA to treat glenohumeral osteoarthritis, massive rotator cuff tears, proximal humerus fractures (PHFs), inflammatory arthritis, and other complex shoulder conditions.^20^^,^^28^^,^^30^^,^^38^^,^^59^

PHFs are among the most notable of the expanded indications for rTSA. These injuries account for approximately 5% of all fractures, with a reported incidence of 82 per 100,000, and are expected to continue rising in prevalence.^22^^,^^31^^,^^43^^,^^44^ Predominantly occurring as fragility fractures in elderly and/or osteoporotic patients, the management of PHFs harbors historical challenges.^2^^,^^47^ Open reduction and internal fixation has been reported to have nonunion or malunion rates of up to 34% and complication rates approaching 44%.^6^ This commonly includes postoperative symptomatic hardware and stiffness.^6^ rTSA has emerged as an alternative to open reduction and internal fixation for treating PHFs among older patients, steadily outpacing other options like hemiarthroplasty and anatomic total shoulder arthroplasty. Both hemiarthroplasty and anatomic total shoulder arthroplasty have decreased in use for PHFs.^3^^,^^38^^,^^53^ Additionally, current evidence backs superior outcomes in PHFs treated with rTSA, characterized by reduced failure rates, improved cost-effectiveness, and greater patient satisfaction.^1^^,^^13^^,^^33^^,^^62^

Despite these trends, uncertainty persists in understanding how rTSA outcomes for PHF compare to those for RCA.^4^ Although comparisons with RCA exist in the rTSA literature, studies directly evaluating PHF versus RCA are small in isolation, inconsistent, and seldom aggregated, limiting their clinical applicability.^42^^,^^50^ The aim of this study is to systematically compare clinical and functional outcomes between patients undergoing rTSA for PHFs and RCA.

## Methods

### Study creation and initial search

This study was performed under the guidelines of the most recent Preferred Reporting Items for Systematic Reviews and Meta-Analyses and was pre-registered in the PROSPERO registry for systematic reviews and meta-analyses (CRD420251110625).^41^ PubMed, CINAHL, MEDLINE, and Web of Science electronic databases were queried from database inception through July 25, 2025, for studies reporting clinical outcomes of rTSA for PHFs versus RCA. The search terms used were (“reverse” AND “shoulder” AND [“arthroplasty” OR “replacement”] AND “fracture” AND [“humerus” OR “humeral”] AND “proximal” AND “cuff” AND “arthropathy”).

### Inclusion and exclusion criteria

Inclusion criteria were prospective, retrospective, or other studies that were composed of entirely adult patients (≥18 years) affected by either PHFs or RCA and having then undergone rTSA. Studies must have reported postoperative information on patient-reported outcome measures (PROMs), ROM, and/or surgical details. Exclusion criteria were cadaveric studies, studies with pediatric patients or pediatric-containing data, nonoperative-only papers, large database/registry studies lacking outcome granularity, non-English studies, studies with cohort sizes of 4 or less (to avoid data skewing and promote generalizability), case reports, letters, surveys, systematic reviews, meta-analyses, and publications without full texts.

### Article screening process

All articles that were gathered from the initial database query were uploaded into Rayyan, a public website used for systematic reviews.^40^ One independent screener performed a manual deduplication of articles, followed by article screening based on title and abstract (B.S.): a process from which the results were confirmed by a report from a second independent screener (S.Y.). Once any disagreements were resolved by the first author, full-text screening based on inclusion and exclusion criteria was conducted.

### Data extraction

The extraction process was completed by 2 independent authors and included the last name of the first author, year of publication, study inclusion/exclusion criteria, number of patients per cohort, sex, age, follow-up, PROMs, ROM details, surgical and operative outcomes, and any other relevant qualitative and/or quantitative data for narrative reporting and/or statistical analysis. Arising extraction conflicts were resolved by the first author.

### Article quality grading

Due to the nature of this systematic review, all observational studies included were inherently classified as “comparative” to proceed with the appropriate grading of their quality using the Methodological Index for Non-Randomized Studies (MINORS) scale.^56^ They were graded out of 24 points on a 12-item spectrum, with each item rated from 0 to 2 points. All studies were either considered “high quality,” “moderate quality,” or “low quality” based on their scoring and inherent comparative nature, following prior systematic review precedent.^32^ High-quality articles scored 24 points, moderate-quality articles scored 15–23 points, and low-quality articles scored less than 15 points.^32^

### Certainty assessment

Outcome certainty was assessed using the Grading of Recommendations, Assessment, Development, and Evaluation system.^18^ This allotted the labeling of outcome certainty as “very low,” “low,” “moderate,” or “high” based on study-specific factors. Should a study be observational, the standard approach is to begin outcome labeling at “low” certainty and progress accordingly due to presumed limitations associated with a lack of randomization in such studies.

### Statistical analyses

Microsoft Excel for Microsoft 365 Apps for Enterprise, version 2505 (Redmond, WA: Microsoft Corporation) and the IBM Statistical Package for the Social Sciences (SPSS), version 30.0 (Armonk, NY: IBM Corporation) were utilized for statistical analyses. Denoted averages are representative of frequency-weighted means (FWMs), standard deviations (SDs), pooled SDs, and other descriptive statistics such as percentages and ranges for further defining data in instances of reporting heterogeneity. If quantitative statistics were not appropriate for analyzing collated data, a qualitative/narrative approach was employed. Furthermore, a random effects meta-analysis was performed using unstandardized mean differences (MDs) or risk ratios with 95% confidence intervals (CIs) for continuous or binary variables, respectively, when appropriate. In the case of a study presenting with at least one zero-event, a continuity correction of 0.5 was added to all values in the study by SPSS to promote appropriate meta-analysis practices.^16^ For estimating the conversion of ranges to SDs during data synthesis, an extrapolated approach to the standards described by Hozo et al (2005) was adopted.^21^ When cohort data were further divided by subgroup, an FWM and pooled SD approach was utilized to position data for homogenous analysis.

## Results

### Initial search results

**Figure 1:**
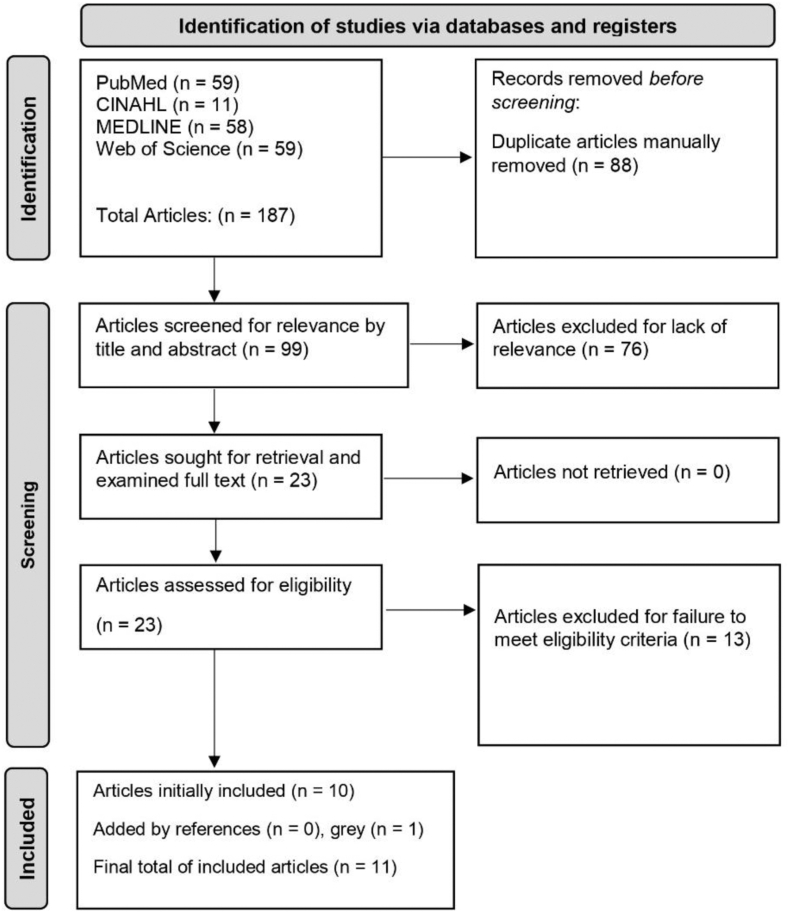
The Preferred Reporting Items for Systematic Reviews and Meta-Analyses (PRISMA) diagram, outlining this study's progression of the search algorithm.

The search algorithm identified a total of 187 articles; after manual deduplication, only 99 remained. Screening by title and abstract yielded 23 articles to be sought for retrieval, of which all were retrieved and 10 were included for final analysis. One additional article was yielded from the gray literature search, and none from the reference screening. The culmination of this process uncovered 11 total articles for data extraction (Fig. 1).^7^^,^^12^^,^^27^^,^^34^^,^^35^^,^^37^^,^^49^^,^^54^^,^^57^^,^^58^^,^^60^

### Article quality results

Ten of the included articles were retrospective comparative cohort studies, with Sebastia-Forcada et al (2020) representing the only prospective comparative cohort study.^54^ The mean overall MINORS score was 18.2 ± 2.4 (out of 24 points); the mean MINORS score for the retrospective studies (n = 10) was 17.7 ± 1.8, and the mean MINORS score for the prospective studies (n = 1) was 23 ± 0. Ultimately, all included studies were observational and of moderate quality (Table I). Conservatively, the certainty of all outcomes was downgraded to “very low” to account for potential risks of bias pertaining to the usage of observational data, alongside suspected heterogeneity-related imprecision.

**Table I:** The Methodological Index for Non-Randomized Studies grading for all included articles.

Author (yr)	Total MINORS score	Clearly stated aim	Inclusion of consecutive patients	Prospective collection of data	End points appropriate to study aim	Unbiased assessment of study end point	Follow-up period appropriate to study aim	Less than 5% lost to follow-up	Prospective calculation of the study size	Adequate control group	Contemporary groups	Baseline equivalence of groups	Adequate statistical analysis
Barnett (2025)^7^	19	2	2	0	2	1	2	2	0	2	2	2	2
Bolam (2024)^12^	19	2	2	2	2	1	2	1	0	2	2	1	2
Keller (2023)^27^	18	2	2	0	2	1	2	2	0	2	2	1	2
Lindbloom (2019)^34^	15	2	2	0	2	1	2	0	0	2	2	1	1
Maier (2023)^35^	15	2	2	0	1	1	2	0	0	2	2	1	2
Matar (2020)^37^	17	2	2	2	2	1	2	0	0	2	2	0	2
Roddy (2021)^49^	17	2	2	2	2	1	2	0	0	2	2	1	1
Sebastia-Forcada (2020)^54^	23	2	2	2	2	2	2	2	2	2	2	1	2
Tan (2023)^57^	20	2	2	0	2	1	2	1	2	2	2	2	2
Vaccaro (2025)^58^	20	2	2	0	2	1	2	1	2	2	2	2	2
Wanga (2023)^60^	17	2	0	0	2	1	2	2	0	2	2	2	2

*MINORS*, Methodological Index for Non-Randomized Studies.

### Patient and study characteristics

A total of 1,832 patients underwent rTSA for PHFs (FWM age = 73.6 ± 8.6 years [72.7% reporting]; follow-up = 4.5 ± 2.7 years [63.6% reporting]; 85.4% female [90.9% reporting]; body mass index 30.3 ± 6.9 kg/m^2^ [45.5% reporting]) and 4,866 for RCAs (FWM age = 73.2 ± 7.6 years [72.7% reporting]; follow-up = 5.4 ± 3.2 years [63.6% reporting]; 60.3% female [90.9% reporting]; body mass index 29.9 ± 4.7 kg/m^2^ [45.5% reporting]) (Table II). Most authors (81.8%; n = 9) forewent RCA inclusion/exclusion detailing. However, 54.5% of authors (n = 6 studies) elected to either explicitly exclude fracture sequelae (nonunion and/or malunion) from their PHF-indicated cohorts entirely or separate them into a unique subcohort to be compared with all other pathologies being studied: a subcohort that was electively excluded from the present systematic review and synthesis to conform to the majority author-trend of excluding such fracture sequelae for homogeneity purposes.^12^^,^^34^^,^^49^^,^^54^^,^^57^^,^^58^ Consideringly, it should be noted that Barnett et al (2025) was the only study that elected to pool these fracture sequelae with the other PHF data, though of only minority population and effect (Table II).^7^

**Table II:** Demographic and cohort details table for included studies.

Author (yr)	Diagnosis group	Diagnosis group inclusion/exclusion details	Cohort size (number)	Average age (SD/range) in years	Average follow-up (SD/range) in years	Female number (%)	Body mass index (BMI) (SD/range) in kg/m^2^
Barnett (2025)^7^	PHF	Included 4-part fractures, 2-part fractures, nonunion, malunion, and post-traumatic avascular necrosis	23	69.4 (9.3)	2.7 (1.2)	20 (87.0%)	33.7 (12.2)
	RCA	-	123	68.6 (9.1)	3.3 (1.5)	68 (55.3%)	30.5 (5.9)
Bolam (2024)^12^	PHF	Included only acute fractures that occurred within 3 mo of injury and excluded nonunion/malunion instances	740	76.0 (8.6)	4.0 (3.0)	633 (85.5%)	-
	RCA	-	3,051	73.6 (7.4)	4.9 (3.5)	1,620 (53.1%)	-
Keller (2023)^27^	PHF	-	21	70.7 (8.6)	2.9 (0.6)	16 (76%)	30.0 (6.7)
	RCA	-	27	65.3 (12.1)	3.1 (0.6)	17 (63%)	31.3 (7.6)
Lindbloom (2019)^34^	PHF	Included only fractures that underwent treatment within 6 weeks of injury, separated nonunion/malunion	18	-	>2 yr	11 (61.1%)	-
	RCA	-	221	-	>2 yr	133 (60.2%)	-
Maier (2023)^35^	PHF	Excluded all acute fractures that occurred within 30 d of operation	70	71.4 (7.9)	4.7 (1.9)	56 (80.0%)	-
	RCA	-	119	72.1 (6.8)	7.4 (2.5)	77 (64.7%)	-
Matar (2020)^37^	PHF	-	12	-	>1 yr	-	-
	RCA	-	94	-	>1 yr	-	-
Roddy (2021)^49^	PHF	Included fractures that occurred within 6 weeks of operation, separated nonunion/malunion	25	73 (9)	2 (1-5)	21 (84%)	26 (23-28)
	RCA	-	225	69 (10)	2 (1-5)	124 (55.1%)	29 (26-34)
Sebastia-Forcada (2020)^54^	PHF	Included fractures that were treated within 7 d of injury that were defined as not amenable to reconstruction, as well as displaced 3-part and 4-part fractures	67	73.5 (6.9)	8.4 (5-11)	52 (77.6%)	30.4 (3.3)
	RCA	Included rotator cuff tears in the setting of end-stage arthritis as well as pseudoparalysis and loss of arm elevation due to massive rotator cuff tear both with and without glenohumeral arthritis	64	70.6 (6.1)	8.4 (5-11)	43 (67.2%)	31.3 (3.7)
Tan (2023)^57^	PHF	Included minimum 3-part or comminuted fracture that was operated on within 6 weeks of injury, excluding nonunion/malunion	15	75.4 (5.8)	>1 yr	11 (73.3%)	-
	RCA	Included cases with radiographic evidence of glenohumeral osteoarthritis and massive rotator cuff tear	60	72.8 (4.8)	>1 yr	38 (63.3%)	-
Vaccaro (2025)^58^	PHF	Included fractures that occurred within 4 weeks that were 3-part or 4-part in nature, separated nonunion/malunion	39	71.6 (7)	9.5 (1.3)	26 (66.7%)	30.9 (9)
	RCA	-	50	72.5 (6)	9.7 (1.2)	32 (64%)	29.6 (8)
Wanga (2023)^60^	PHF	-	802	69.3% of cohort between 70 and 80 yr	>5 yr	707 (88.2%)	39.9% of cohort over 30 BMI
	RCA	-	802	69.3% of cohort between 70 and 80 yr	>5 yr	707 (88.2%)	39.9% of cohort over 30 BMI

*PHF*, proximal humerus fracture; *RCA*, rotator cuff arthropathy; *SD*, standard deviation.

A “-” was used to denote a metric that was unspecified within the respective study.

### Functional and patient-reported outcomes

**Figure 2:**
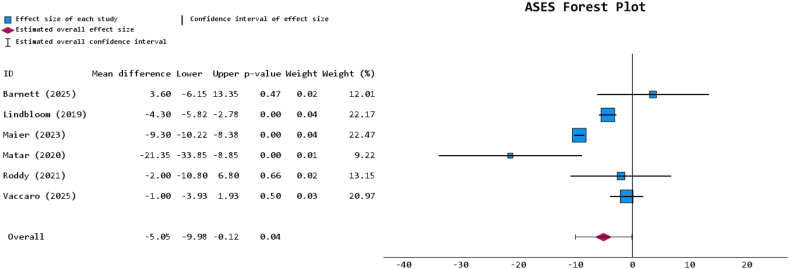
Forest plot for postoperative ASES comparing PHF- versus RCA-indicated rTSA of reporting studies. *ASES*, American Shoulder and Elbow Surgeons; *PHF*, proximal humerus fracture; *RCA*, rotator cuff arthropathy; *rTSA*, reverse total shoulder arthroplasty.

The FWM postoperative visual analog scale (VAS)-pain and American Shoulder and Elbow Surgeons (ASES) scores for the PHF-indicated cohort were 2.3 ± 2.0 and 73.9 ± 13.4 (both 54.5% reporting), respectively; and for the RCA-indicated cohort were 1.9 ± 2.1 and 80.2 ± 18.6 (both 54.5% reporting), respectively (Table III). The PHF-indicated cohort did not present with a statistically significant change in VAS-pain score compared to the RCA-indicated cohort (*P* = .621) via meta-analysis of 6 studies. ASES scores were significantly lower in the PHF-indicated cohort per the meta-analysis of the respective 6 studies (*P* = .045; MD = −5.053 [95% CI: −9.982, −0.124]) (Fig. 2). Qualitatively, 8 of the 11 studies reported 13 different instances of additional heterogeneous PROM metrics, of which the RCA-indicated cohort demonstrated either marginally or significantly superior outcomes in 9 instances (69.2%): details further granularized in Table III. Tandemly, in the 4 studies that reported on patient satisfaction aside from the aforementioned PROMs, 3 (75%) reported either marginally or significantly higher satisfaction in the RCA-indicated cohort (Table III).

**Table III:** Functional and patient-reported outcomes table for included studies.

Author (yr)	Diagnosis group	Average VAS-pain score (SD) at final follow-up	VAS *P* value	Average ASES score (SD) at final follow-up	ASES *P* value	Other average PROMs score (SD) at final follow-up	Additional PROM *P* value	Independent satisfaction description at final follow-up	Independent satisfaction *P* value
Barnett (2025)^7^	PHF	1.8 (3.5)	*P* = .886	80.7 (20.7)	*P* = .777	Single Assessment Numerical Evaluation (SANE): 88.3 (4.1); Simple Shoulder Test (SST): 9.0 (1.2)	SANE: *P* = .377; SST: *P* = .366	-	-
	RCA	1.7 (2.5)		77.1 (22.2)		SANE: 80.7 (20.7); SST: 7.7 (3.2)		-	-
Bolam (2024)^12^	PHF	-	-	-	-	Oxford Shoulder Score (OSS): 31.1 (0.5)	OSS: *P* < .001	-	-
	RCA	-		-		OSS: 35.6 (0.2)		-	-
Keller (2023)^27^	PHF	-	-	-	-	Quick Disabilities of the Arm, Shoulder, and Hand (qDASH): 31.0 (16.7)	qDASH: *P* = .005	-	-
	RCA	-		-		qDASH: 18.4 (10.6)		-	-
Lindbloom (2019)^34^	PHF	-	-	66.9 (8.8)	-	SST: 6.0 (1.1)	-	Patient-Rated Satisfaction Score: 6.8 (1.0)	-
	RCA	-		71.2 (2.2)		SST: 5.8 (0.3)		Patient-Rated Satisfaction Score: 8.0 (0.25)	-
Maier (2023)^35^	PHF	1.1 (2.1)	*P* = .7	73.3 (66.6-80.0)	*P* = .0022	Shoulder Pain and Disability Index (SPADI)-Total: 31.0 (24.9-37.2)	SPADI: *P* = .0001	-	-
	RCA	0.6 (1.5)		82.6 (76.6-88.5)		SPADI-Total: 19.5 (13.1-25.9)		-	-
Matar (2020)^37^	PHF	3.5 (3.73)	-	54.17 (22.03)	-	-	-	-	-
	RCA	1.84 (2.47)		75.52 (20.65)		-		-	-
Roddy (2021)^49^	PHF	-	-	71 (24)	*P* = .64	-	-	96%	-
	RCA	-		73 (21)		-		92%	-
Sebastia-Forcada (2020)^54^	PHF	4.2 (1.2)	*P* = .062	-	-	University of California Los Angeles (UCLA): 24.6 (7.4); qDASH: 28.0 (7.6); Constant Murley score (CMS): 48.6 (14.0)	UCLA: *P* = .088; qDASH: *P* = .135; CMS: *P* = .125	VAS-satisfaction: 6.2 (1.5)	*P* = .002
	RCA	4.7 (1.8)		-		UCLA: 26.6 (5.9); qDASH: 26.1 (6.9); CMS: 52.3 (12.5)		VAS-satisfaction: 7.0 (1.4)	
Tan (2023)^57^	PHF	1.1 (1.8)	*P* = .343	-	-	UCLA: 25.3 (5.1); OSS: 22.5 (10.9); CMS: 51.8 (13.1)	UCLA: *P* = .211; OSS: *P* = .266; CMS: *P* = .124	73.3% good or better satisfaction	-
	RCA	1.8 (2.6)		-		UCLA: 27.3 (5.7); OSS: 19.4 (9.2); CMS: 58.3 (14.7)		83.3% good or better satisfaction	
Vaccaro (2025)^58^	PHF	1.8 (1)	-	82 (7)	-	SANE: 78 (9)	-	-	-
	RCA	1.6 (1)		83 (7)		SANE: 80 (8)		-	
Wanga (2023)^60^	PHF	-	-	-	-	-	-	-	-
	RCA	-		-		-		-	

*VAS*, visual analog scale; *PROMs*, patient-reported outcome measures, *PHF*, proximal humerus fracture; *RCA*, rotator cuff arthropathy; *SD*, standard deviation; *ASES*, American Shoulder and Elbow Surgeons.

A “-” was used to denote a metric that was unspecified within the respective study.

### Rehabilitation and ROM results

**Figure 3:**
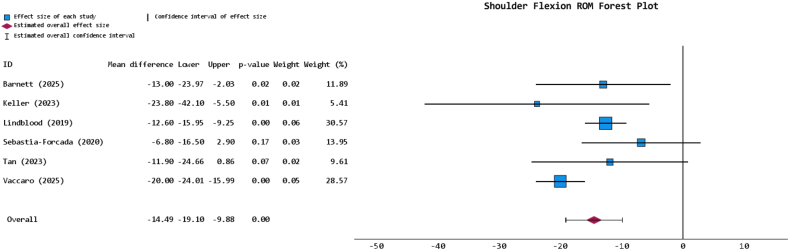
Forest plot for shoulder flexion ROM comparing PHF- versus RCA-indicated rTSA of reporting studies. *ROM*, range of motion; *PHF*, proximal humerus fracture; *RCA*, rotator cuff arthropathy; *rTSA*, reverse total shoulder arthroplasty.

**Figure 4:**
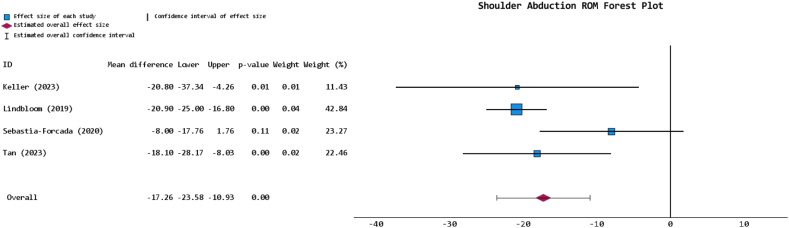
Forest plot for shoulder abduction ROM comparing PHF- versus RCA-indicated rTSA of reporting studies. *ROM*, range of motion; *PHF*, proximal humerus fracture; *RCA*, rotator cuff arthropathy; *rTSA*, reverse total shoulder arthroplasty.

The rehabilitation protocol was explicitly reported in 4 studies, across all of which the PHF- and RCA-indicated management strategies were either identical or near-identical, with the shoulder immobilized for anywhere between 2 and 6 weeks before ROM or weight-bearing exercises began (Table IV). The FWM shoulder flexion for the PHF-indicated cohort was 117.0° ± 27.2° (54.5% reporting) and for the RCA-indicated cohort was 136.0° ± 15.8° (54.5% reporting). Meta-analysis of the reporting studies revealed that the PHF-indicated cohort had significantly reduced shoulder flexion ROM (*P* < .001; MD = −14.491 [95% CI: −19.1, −9.881]) at final follow-up (Fig. 3). The RCA-indicated cohort had either marginally or significantly greater external rotation and internal rotation in 83.3% (n = 5 out of 6 reporting studies) and 66.7% (n = 4 out of 6 reporting studies) of instances, respectively (Table IV). These rotational metrics were not meaningfully analyzed beyond such narrative reporting due to marked heterogeneity, as some studies reported values in degrees and others in vertebral level indications or Constant-Murley scores.^14^^,^^57^ The FWM shoulder abduction for the PHF-indicated cohort was 98.9° ± 27.8° (36.4% reporting), for the RCA-indicated cohort this was 119.8° ± 16.0° (36.4% reporting), and meta-analysis revealed that the PHF-indicated cohort had a significantly reduced shoulder abduction ROM at final follow-up (*P* < .001; MD = −17.257 [95% CI: −23.582, −10.993]) (Fig. 4).

**Table IV:** Rehabilitative outcomes table for included studies.

Author (yr)	Diagnosis group	Rehabilitation protocol	Average flexion (SD/range) in degrees at final follow-up	Flexion *P* value	Average external rotation (ER) (SD/range in degrees or vertebral level/value, or CMS at final follow-up	ER *P* value	Average internal rotation (IR) (SD/range) in degrees, vertebral level/value, or CMS at final follow-up	IR *P* value	Average abduction (SD/range) in degrees at final follow-up	Abduction *P* value
Barnett (2025)^7^	PHF	-	128.9 (38.8)	*P* = .0242	45.9 (18.9)	*P* = .0679	L5	*P* = .632	-	-
	RCA	-	141.9 (21.1)		40.3 (11.7)		L5		-	
Bolam (2024)^12^	PHF	-	-	-	-	-	-	-	-	-
	RCA	-	-		-		-		-	
Keller (2023)^27^	PHF	A shoulder immobilizer was used within the first 2 weeks. From weeks 2-6, patients were allowed to perform non-weight-bearing ROM exercises. Weight bearing proceeded by weeks 6-16, with a gradual return to full activity at weeks 16-24	106.9 (35.7)	*P* = .01	42.4 (23.2)	*P* = .18	39.5 (17.7)	*P* = .002	102.9 (33)	*P* = .02
	RCA	Identical to the PHF cohort, except weight-bearing of up to 5 pounds was allowed from weeks 2-6	130.7 (29.0)		50.6 (17.9)		55 (14.5)		123.7 (25.5)	
Lindbloom (2019)^34^	PHF	-	129.4 (21.43)	-	45.3 (22.0)	-	4.0 (1.21)	-	106.3 (27.2)	-
	RCA	-	142.0 (4.1)		49.5 (4.2)		4.6 (0.25)		127.2 (4.6)	
Maier (2023)^35^	PHF	-	-	-	-	-	-	-	-	-
	RCA	-	-		-		-		-	
Matar (2020)^37^	PHF	-	-	-	-	-	-	-	-	-
	RCA	-	-		-		-		-	
Roddy (2021)^49^	PHF	An abduction sling was used to immobilize patients for the first 6 weeks, after which formal physical therapy was begun	-	-	-	-	-	-	-	-
	RCA	Identical to the PHF cohort	-		-		-		-	
Sebastia-Forcada (2020)^54^	PHF	A single sling was used to immobilize patients for the first 2 weeks, which was then followed with a minimum of 4 weeks of physiotherapy	113.6 (28.4)	*P* = .167	5.8 (2.1)	*P* = .113	3.2 (2.1)	*P* = .384	98.4 (27.6)	*P* = .109
	RCA	Identical to the PHF cohort	120.4 (28.2)		6.4 (1.8)		3.6 (2.3)		106.4 (29.4)	
Tan (2023)^57^	PHF	-	105.3 (24)	*P* = .077	4.8 (3.3)	*P* = .051	4.5 (2.9)	*P* = .350	86.7 (19.8)	*P* = .001
	RCA	-	117.2 (22.2)		6.8 (3.6)		3.8 (2.5)		104.8 (17.3)	
Vaccaro (2025)^58^	PHF	A shoulder sling was used in the first 4 weeks, after which active exercises began at weeks 6-8	120 (9)	*P* < .001	20 (6)	*P* < .001	33 (10)	-	-	-
	RCA	Identical to the PHF cohort	140 (10)		26 (7)		35 (8)		-	
Wanga (2023)^60^	PHF	-	-	-	-	-	-	-	-	-
	RCA	-	-		-		-		-	

*ROM*, range of motion; *PHF*, proximal humerus fracture; *RCA*, rotator cuff arthropathy; *CMS*, Constant Murley score; *SD*, standard deviation.

A “-” was used to denote a metric that was unspecified within the respective study.

### Operative outcomes and details

In the 7 studies where surgical approach was specified, the deltopectoral approach was either the majority or the sole approach to rTSA for both PHF- (100%) and RCA-indicated cohorts (100%) (Table V). All implants specified were stemmed (100%; 36.4% reporting). And an either majority or entirely cementless technique was employed in 3 of 4 reporting studies, though humeral cementing trended toward a majority in several studies: data further detailed in Table V. Qualitatively, of the 6 studies that reported implant survival rate, 5 (83.3%) reported a comparatively higher long-term rate for the PHF-indicated cohort. The pooled revision rate for the PHF-indicated cohort was 3.2% compared to the RCA-indicated cohort at 3.8% (both 81.2% reporting), and meta-analysis revealed no significant risk of revision difference between groups (*P* = .630). By etiology, dislocation (38.5%), periprosthetic fracture (19.2%), infection (15.4%), and aseptic loosening (15.4%) constituted the largest proportions in descending order of detailed revisions in the PHF-indicated cohort. In the RCA-indicated cohort, this corresponded to aseptic loosening (27.1%), dislocation (25.2%), and infection (20.6%), respectively (Table V). Revision etiology subgroup analysis of reporting studies revealed no significant differences in risk of infection (*P* = .62; 36.4% reporting), periprosthetic fracture (*P* = .26; 54.5% reporting), or aseptic loosening (*P* = .52; 27.3% reporting); however, a significantly higher risk of revision due to dislocation was observed in the PHF-indicated cohort (*P* = .04; risk ratio = 1.59 [95% CI: 1.02, 2.48]; 45.5% reporting).

**Table V:** Operative outcomes and details table for included studies.

Author (yr)	Diagnosis group	Surgical approach	Stem use	Cement use	Implant survival rate (95% confidence interval) and details	Revisions (%)	Revision details
Barnett (2025)^7^	PHF	-	-	-	2 yr: 100% 5 yr: 86.9%	3 (13.0%)	(2) periprosthetic fracture, (1) pain
	RCA	-	-	-	2 yr: 99.1% 5 yr: 91.7%	3 (2.4%)	-
Bolam (2024)^12^	PHF	Majority deltopectoral	-	0.5% glenoid cementing, 36.4% humeral cementing	1 yr: 99.0% (99.3-99.7) 5 yr: 97.7% (96.4-99.0) 10 yr: 97.3% (95.8-98.8)	14 (1.9%)	(3) infection, (4) aseptic loosening, (1) periprosthetic fracture, (6) dislocation
	RCA	Majority deltopectoral	-	0.2% glenoid cementing, 34.2% humeral cementing	1 yr: between 98-100% 5 yr: between 96-98% 10 yr: between 92-93%	112 (3.7%)	(27) infection, (39) aseptic loosening, (6) periprosthetic fracture, (9) mechanical failure, (29) dislocation, (4) notching or erosion, (14) pain
Keller (2023)^27^	PHF	Deltopectoral	All stemmed	48% humeral cementing	3 mo: 95.2% 6 mo: 95.2% 1 yr: 90.5% 2-4 yr: 90.5%	2 (9.5%)	(2) dislocation
	RCA	Deltopectoral	All stemmed	7% humeral cementing	3 mo: 96.3% 6 mo: 96.3% 1 yr: 92.6% 2-4 yr: 85.2%	4 (15%)	(1) dislocation, (1) aseptic loosening, (2) periprosthetic fracture
Lindbloom (2019)^34^	PHF	Deltopectoral	-	-	-	0 (0%)	-
	RCA	Deltopectoral	-	-	-	2 (0.9%)	(1) instability, (1) dislocation
Maier (2023)^35^	PHF	-	-	-	-	-	-
	RCA	-	-	-	-	-	-
Matar (2020)^37^	PHF	-	-	-	-	-	-
	RCA	-	-	-	-	-	-
Roddy (2021)^49^	PHF	Deltopectoral	All stemmed	-	3 yr: 97% (81-100)	2 (8%)	(2) dislocation
	RCA	Deltopectoral	All stemmed	-	3 yr: 95% (90-97)	12 (5.3%)	(7) dislocation, (2) infection, (3) periprosthetic fracture
Sebastia-Forcada (2020)^54^	PHF	Deltopectoral	All stemmed	All cementless	10 yr: 96.9% (91.1-99.9)	1 (1.5%)	(1) periprosthetic fracture
	RCA	Deltopectoral	All stemmed	All cementless	10 yr: 85.1% (76.7-99.9)	5 (7.8%)	(1) periprosthetic fracture, (1) dislocation, (2) aseptic loosening, (1) infection
Tan (2023)^57^	PHF	-	-	-	-	0 (0%)	-
	RCA	-	-	-	-	0 (0%)	-
Vaccaro (2025)^58^	PHF	Deltopectoral	All stemmed	All humeral cemented	-	4 (10.3%)	(2) instability, (1) infection, (1) periprosthetic fracture
	RCA	Deltopectoral	All stemmed	All humeral cemented	-	4 (8%)	(2) instability, (2) infection
Wanga (2023)^60^	PHF	-	-	-	5 yr: 96%	33 (4.1%)	-
	RCA	-	-	-	5 yr: 95%	42 (5.2%)	-

*PHF*, proximal humerus fracture; *RCA*, rotator cuff arthropathy.

A “-” was used to denote a metric that was unspecified within the respective study.

## Discussion

This systematic review and meta-analysis provides a comprehensive comparison of clinical outcomes following rTSA performed for PHF versus its benchmark indication, RCA. As rTSA use continues to rise and indications broaden, understanding differences in functional and revision outcomes based upon surgical indication is critical to patient counseling and ongoing clinical research pertaining to rTSA. Our pooled analysis shows that PHF-indicated rTSA is associated with modestly lower ASES scores, reduced forward flexion and abduction, and a higher risk of dislocation-related revision compared with RCA. Other patient-reported and ROM measures, while not always statistically significant or able to undergo meta-analysis, generally favored RCA. Together, these findings underscore the need to optimize techniques and identify risk factors unique to PHF rTSA.

PROMs consistently favored the RCA-indicated rTSA cohort, and while VAS-pain scores were not significantly different between groups, the present study identified a significantly lower PHF-indicated ASES score by an MD of approximately 5 points. Although this disparity may raise comparative concerns, it can be better understood via contextualization within existing literature. First, while the 5-point difference reached statistical significance, in many cases it may be considered below the Minimally Clinically Important Difference for rTSA-specific ASES pre- to post-operative gains, which has been reported to be around 10-11 points.^9^^,^^55^ This suggests that the practical effect of this ASES difference between the RCA- and PHF-indicated cohorts may be less clinically relevant. Additionally, many studies do not consistently report preoperative ASES values in such comparative analyses, and thus, baseline function and scores may be confounding findings. Studies have documented markedly higher preoperative ASES scores for RCA-indicated rTSA compared to PHF-indicated, with some case differences approaching 20-point differences.^25^^,^^26^^,^^52^ This discrepancy is not unexpected, given the acute traumatic underpinning of PHFs that can produce more immediate functional compromise than degenerative conditions. While patients with PHFs can achieve substantial gains from pre- to- post-operative status, the multifactorial nature of the ASES score may still reflect a significant difference when compared to RCA-indicated cohorts that often begin at higher baseline ASES.^57^ Further investigation into the mechanisms underlying post-rTSA shoulder function disparity also suggests that greater tuberosity healing may be implicated. In PHFs, it has been hypothesized that compromised bone stock and vascularity make it more challenging to optimize humeral inclination angle necessary for promoting adequate tuberosity healing and achieving optimal postoperative shoulder function.^39^^,^^61^ Furthermore, multicenter studies have shown that PHF-indicated rTSAs often produce poorly healed tuberosities if not specifically accounted for.^36^ However, these factors remain hypotheses, and differences may reflect etiologically complex, incompletely understood mechanisms including chronic adaptation in long-standing cuff insufficiency versus acute trauma-related soft tissue changes following PHF: all with additional potential for pathophysiological overlap between indications. Nonetheless, this review quantifies the observed ASES disparity, placing it within the context of current evidence and at a higher level of analysis than previously reported.

Comparison of postoperative ROM findings mirrored the ASES results, with the PHF-indicated cohort demonstrating significantly reduced shoulder flexion and abduction by MDs of 14 and 17°, respectively, alongside repeatedly reduced rotation metrics as synthesized narratively across reporting studies. Similar to the rationale provided for the ASES score and overall shoulder function disparity, greater tuberosity healing has also been linked to improved shoulder ROM after rTSA and may likewise contribute to the limitations observed in the PHF-indicated cohort. The literature, however, presents some nuance on the matter; certain studies report significant differences in metrics such as abduction, others no such difference in forward flexion, for example.^23^^,^^39^^,^^51^ From a muscular standpoint, surface electromyography following rTSA has shown significantly increased middle deltoid activation during abduction, flexion, and external rotation.^48^ Additionally, computation models on this matter have suggested that in patients with RCA, the chronically degenerated rotator cuff promotes heightened compensatory middle deltoid activation, which when combined with the muscular demands of rTSA, may help to explain the superior ROM outcomes observed in the RCA-indicated cohort.^45^ Moreover, studies of PHFs have also shown that a considerable proportion of treated cases have concomitant axillary nerve injuries, particularly in more complex fractures, and so it would not be implausible to presume some degree of diminished middle deltoid activation and impairment to deltoid-reliant ROM outcomes.^46^ Importantly, just as tuberosity healing was not examined by included studies in the present review, neither were muscle fiber recruitment patterns nor nerve-injury profiles, delineating potentially insightful and novel avenues for subsequent studies to more comprehensively profile these post-rTSA ROM discrepancies.

When examining revisions, overall rates remained low at under 4% for both the PHF- and RCA-indicated cohorts individually. However, meta-analysis revealed a notable exception, as the PHF-indicated cohort showed a significantly increased risk of revision specifically due to dislocation. Dislocation is a well-documented complication in rTSA literature, and improper deltoid tensioning has been purported as one key mechanism contributing to instability and consequent dislocation and revision surgery.^5^^,^^17^ Achieving appropriate deltoid tension during rTSA surgery is often reliant on the finalized humeral length and implant, which can be a particularly challenging and multifactorial decision to make in PHF cases where humeral bone loss can complicate the restoration of humeral length-nativity.^10^^,^^24^ In these instances, potentially inadequately distalized humeral components can then predispose PHF-indicated rTSA patients to suboptimal deltoid tensioning, increasing the risk of dislocation and subsequent revision, where re-tensioning is then correctively performed.^24^^,^^29^ While this mechanism may explain the observed phenomenon of increased dislocation revision in the PHF-indicated cohort, soft tissue compromise has also been implicated as an additional contributing factor.^29^ Nevertheless, as with other aforementioned rationalizations, the included studies did not provide further granularization and exploration of these processes, and so such considerations remain conjectures for discussion and future investigation.

The study's findings must be interpreted within the context of its limitations. First, analyses were based primarily on moderate quality observational and limited-outcome-certainty studies, as assessed by MINORS and Grading of Recommendations, Assessment, Development, and Evaluation criteria, respectively, which inherently carries risks of biases and constrains the strength of pooled conclusions. This becomes further complicated in the presence of observed heterogeneity in data defining and reporting, especially regarding rotation ROM, additional PROMs, the use of cement and stemmed implants, inclusion/exclusion criteria specifically in the RCA-indicated cohort, and paucity of nonrevision complication elucidation: a conglomeration that further limits more advanced comparisons by subgrouping and statistical transformation. Of particular importance was heterogeneity among rehabilitation protocols. The absence or variation of rehabilitation protocols in certain studies may have confounded findings.^15^^,^^19^ Lastly, averages, ranges, and SDs were also inconsistently presented, and while this study performed estimations and transformations via peer-reviewed and widely accepted methodologies for pooled analyses, the actions thereof must be acknowledged.

This study remains the largest and most comprehensive inquiry into the comparative outcomes following rTSA in PHF-indicated and RCA-indicated patients. Future high-quality and methodologically rigorous investigations are warranted to better ascribe risk factors and considerations for clinical outcomes following rTSA in variably indicated patients to better understand the uncovered discrepancies. Moreover, the present study highlights that clinical outcomes research pertaining to rTSA should account for variation based upon surgical indications.

## Conclusion

rTSA for PHF yields slightly lower shoulder function and ROM and higher dislocation-related revision risk compared with RCA, though absolute differences are modest. These findings support nuanced pre-operative counseling and highlight opportunities to optimize rTSA-PHF-specific surgical strategies.

## Disclaimers:

Funding: No funding was disclosed by the authors.

Conflicts of interest: Michael C. Fu serves on the Editorial or Governing Board for Arthroscopy and the *HSS Journal* and is a consultant for Stryker. Samuel A. Taylor is a Don Joy Orthopedics - Designer; Honoraria. Joshua S. Dines serves as a Board or Committee Member of ASES, an Arthrex consultant and receives royalties from Arthrex and Conmed Linvatec, research support from Arthrex and HSS, publishing royalties from Thieme Inc. and Lippincott Williams & Wilkins, and is on the Board of Directors of Viewfi. David M. Dines is a Board or Committee Member for ASES and AAOS, receives publishing royalties from Thieme Inc., is a paid Consultant for Wright Medical Technology, is on the Editorial Board for Springer, serves as a Zimmer Biomet Inc. Consultant and receives royalties for the design and invention of bi-modular, total, and comprehensive shoulder replacement systems. Lawrence V. Gulotta has Responsive Arthroscopy ownership interest, has Imagen Inc. ownership interest, serves as Smith & Nephew Speakers' Bureau, and is Zimmer-Biomet, Inc. consultant & Speakers' Bureau, and serves as an Editorial or Governing Board for the *HSS Journal*. Christopher M. Brusalis has received honoraria from Enovis. The other authors, their immediate families, and any research foundations with which they are affiliated have not received any financial payments or other benefits from any commercial entity related to the subject of this article.
